# Association of Length of Time Spent in the United States With Opioid Use Among First-Generation Immigrants

**DOI:** 10.1001/jamanetworkopen.2019.13979

**Published:** 2019-10-25

**Authors:** Brian D. Sites, Matthew A. Davis

**Affiliations:** 1 Department of Anesthesiology and Perioperative Medicine, Dartmouth-Hitchcock Medical Center, Lebanon, New Hampshire; 2 Department of Anesthesiology, Geisel School of Medicine at Dartmouth, Hanover, New Hampshire; 3 Institute for Social Research, University of Michigan, Ann Arbor; 4 Institute for Healthcare Policy and Innovation, University of Michigan, Ann Arbor; 5 University of Michigan School of Nursing, Ann Arbor

## Abstract

This cross-sectional study uses Medical Expenditure Panel Survey data to examine the association of the length of time a first-generation immigrant has spent in the United States with the likelihood of opioid use.

## Introduction

The opioid crisis is unique in that it originated from within the US health care system.^1^ Examining prescription opioid use among immigrants as they spend more time in the United States provides a unique opportunity to quantify the association of the health care system (or other factors) on prescribing practices. If uniquely American cultural factors affect opioid prescribing independent of known sociodemographic and health-related factors, interventions to potentially mitigate the opioid crisis would likely require a more complex and nuanced understanding of traditions and customs.

Therefore, we examined the association of the length of time in the United States with opioid use among first-generation immigrants to evaluate the association of the cultural influence (if any) with opioid prescribing.

## Methods

As this study used only deidentified and publicly available data, it was deemed exempt from institutional board review by the Dartmouth College Committee for the Protection of Human Subjects. We followed the Strengthening the Reporting of Observational Studies in Epidemiology (STROBE) reporting guideline for observational studies.

To examine the association of immigration status (ie, length of time spent in the United States among first-generation immigrants) and prescription opioid use, we conducted a cross-sectional study using data from the Medical Expenditure Panel Survey, a nationally representative health survey of the noninstitutionalized US population sponsored by the Agency for Healthcare Research and Quality. It is a well-known source of nationally representative data on health care expenditures and health service use that includes prescription medications.^2,3^ After aggregating the 2 most recent years of Medical Expenditure Panel Survey data (2014 and 2016) with independent samples, we identified a sample of 13 635 adult immigrants. Based on self-reported length of time spent in the United States, we categorized immigrants as new (<5 years), semiestablished (5 to <15 years), and long-standing (≥15 years) compared with nonimmigrants (born in the United States). Prescription opioid use was identified using a list of established National Drug Codes, and complex survey design methods were used to make national estimates. We examined how opioid use varied across immigration status (including whether immigration occurred as an adult vs child), race/ethnicity, and family income level.

We used logistic regression to adjust for key differences to examine the association between length of time spent in the United States and the odds of prescription opioid use. Data analyses were conducted from February 3, 2019, to April 29, 2019. Analysis was performed using Stata statistical software version 15.1 (StataCorp). *P* values were 2-tailed, and statistical significance was set at .05.

## Results

Among an estimated 41.5 million adult immigrants in the United States, 3.2 million (7.8%) use prescription opioids, with most immigrant opioid users (3.0 million) having arrived in the United States as adults (Table). Nonimmigrants were significantly more likely to use prescription opioids compared with all first-generation immigrants (16.1% vs 7.8%; overall adjusted odds ratio, 1.35; 95% CI, 1.09-1.67; *P* = .005). A positive association was observed between length of time spent in United States and the likelihood of prescription opioid use (*P* for trend < .001) (Figure). Across categories for the length of time spent in the United States, the adjusted rate of opioid use increased from 4.7% (95% CI, 1.1%-8.2%) among new immigrants to 14.8% (95% CI, 12.4%-17.2%) among long-standing immigrants. The adjusted odds of prescription opioid use among long-standing immigrants was more than 4-fold that of new immigrants (adjusted odds ratio, 4.18; 95% CI, 1.76-9.96). Similarly, the odds of opioid use were more than 5-fold higher among nonimmigrants compared with new immigrants (adjusted odds ratio, 5.17; 95% CI, 2.19-12.22).

**Table.** Opioid Use by Immigration Status, Race/Ethnicity, and Income

Characteristic	Total Population, No., Millions	Opioid Users, No. (%), Millions^a^
Immigrant status		
New^b^	2.5	0.1 (2.6)
Semiestablished^c^	11.0	0.6 (5.4)
Long-standing^d^	28.0	2.6 (9.2)
Nonimmigrant	202.3	32.5 (16.1)
Age at immigration^e^		
<18 y	5.8	0.3 (4.3)
≥18 y	35.6	3.0 (8.3)
Race/ethnicity		
Non-Hispanic		
White	156.0	25.8 (16.5)
Black	28.8	4.3 (14.8)
Hispanic	38.3	3.7 (9.7)
Asian	14.2	0.7 (5.0)
Other	7.0	1.3 (18.4)
Family annual income^f^		
Poor or near poor	39.1	7.8 (19.9)
Low	31.0	5.0 (16.2)
Middle	70.5	9.5 (13.5)
High	103.9	13.5 (13.0)

^a^ All estimates weighted to represent the US adult population.

^b^ Lived in the United States less than 5 years.

^c^ Lived in the United States between 5 and 15 years.

^d^ Lived in the United States longer than 15 years.

^e^ Estimated age at immigration based on categories of years in the United States.

^f^ Family annual income was measured as a percentage of the federal poverty level and defined as poor or near poor, less than 125%; low, 125% to less than 200%; middle, 200% to less than 400%; and high, 400% or higher.

**Figure.**
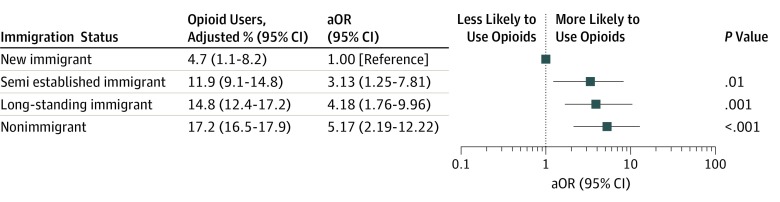
Association of Dose Response of Length of Time Lived in the United States With Prescription Opioid Use Among Adults Values wereadjusted for age (continuous), sex, race/ethnicity (ie, non-Hispanic white, non-Hispanic black, Hispanic, Asian, or other), self-reported pain (ie, mild, moderate, or severe), health insurance status (ie, private, public, or uninsured), physical health composite score (continuous), mental health composite score (continuous), and income as a percentage of the federal poverty level. New immigrants were defined as those who had lived in the United States less than 5 years; semiestablished immigrants, lived in the United States between 5 and 15 years; long-standing immigrants, lived in the United States longer than 15 years; and nonimmigrants, born in the United States. aOR indicates adjusted odds ratio.

## Discussion

Independent of demographic characteristics, health, income, pain perception, and access to health care, we found that as immigrants spent more time in the United States, the likelihood of opioid use increased. Various risk factors for opioid prescribing in the general population, such as race, sex, and mental illness, have been previously described^4^; however, our results potentially point to a unique American culture that promotes opioid use. Although our study was not able to explicitly identify whether assimilation occurred, we suspect that time-sensitive cultural factors may have influenced the dynamic between health care practitioners and immigrant patients as they contemplated the decision to initiate opioid therapy.^5^ Policy efforts to decrease reliance on opioids may benefit from acknowledgment of cultural factors that influence opioid use that are unique to the United States.
